# Modulation of T cell proliferation and cytokine response by Plumbagin, extracted from *Plumbago zeylanica *in collagen induced arthritis

**DOI:** 10.1186/1472-6882-11-114

**Published:** 2011-11-16

**Authors:** Aparanji Poosarla, Rao DN, Rama Rao Athota, Venu Gopal Sunkara

**Affiliations:** 1 Department of Biochemistry, Andhra University, Visakhapatnam, Andhra Pradesh, India; 2 Department of Biochemistry, All Indian Institute of Medical Sciences, New Delhi, India

## Abstract

**Background:**

The extracts of *Plumbago zeylanica *have been used in China and other Asian countries as folk medicine for the treatment of cancer, rheumatoid arthritis and dysmenorrhoea. Effect of Plumbagin (5-hydroxy-2-methyl-1,4-naphthoquinone) purified from *Plumbago zeylanica *on Con A induced T cell proliferation was studied in spleen cells from collagen induced arthritic DBA/1 mice.

**Methods:**

The DBA/1 mice (five per each group) were immunized with 0.1 mL of collagen (emulsified in CFA) by intradermal injection at the base of the tail. On day 20, mice were given a booster dose of collagen (emulsified in IFA) through the same route. Plumbagin was given at different concentrations (3.3, 6.6, 13.3 mg/kg body weight) intraperitoneally. Control mice received olive oil alone. The Con A induced T cell proliferative responses of arthritic and Plumbagin treated mice were studied by cell culture experiments using tritiated Thymidine. In addition the cytokine levels were estimated from the *in vitro *spleen culture supernatants of arthritic mice primed with different concentrations of Plumbagin by ELISA.

**Results:**

Plumbagin enhanced the decreased Con A induced T cell proliferation and Interleukin-2 production in arthritic mice. Moreover elevated levels of IFN- γ were found to be decreased in Plumbagin treated spleen cell culture supernatants. Subclasses of IgG were found to be decreased by Plumbagin treatment, IgG2a reduction seems to be more prominent.

**Conclusion:**

The results obtained in the current study indicate that Plumbagin is very effective in the mechanism based treatment of Rheumatoid arthritis.

## Background

Plumbagin (5-hydroxy-2-methyl-1,4-naphthoquinone), a quinone isolated from the roots of *Plumbago zeylanica *was recently reported to suppress the activation of NF-kappa B in tumor cells [1]. T cells and their cytokine products are the causative factors of autoimmune diseases, including Rheumatoid arthritis. For the induction of arthritis either self reactive T cells must be present in the immunological repertoire or self antigen must be present in conjunction with major histocompatibility complex class II determinants on competent antigen presenting cells. The CD4+ T cell population contains subsets Th1 and Th2 cells, which have been characterized in murine system and have been shown to differ in the secretion of cytokines. Thus Th1 cells can be shown to be the source of IL-2 and Interferon- γ and promote cell mediated immune response. The importance of IFN- γ as a local mediator of inflammation in RA has also been confirmed in collagen induced arthritis. Thus local injection of IFN- γ into the footpads of collagen type II immunized mice accelerates the onset of arthritis and increases the severity of disease [2]. Up regulation of adhesion molecule expression on vascular endothelium by IFN- γ may also promote the homing of lymphocytes and neutrophils to the affected tissues. The quantitative analysis suggests that there are few T cell derived cytokines such as Interleukin-2 (IL-2), Tumour necrosis factor-α (TNF-α), IL-1 and IFN-γ in inflamed synovial tissue.

However, many other cytokines are present in moderate to high concentrations in Rheumatoid arthritis (RA). TNF-α and IL-1 are both present in large quantities in synovial fluid and synovial tissue. The immunohistochemical and mRNA *in situ *hybridization analysis has shown the presence of these cytokines in cells of synovial lining and sublining, including type A synoviocytes and other macrophage like populations [3,4]. To provide a means for homeostasis and downregulation of inflammatory responses, a subclass of cytokines and cytokine receptors are thought to exert anti-inflammatory action in the synovium. There are two TNF receptors, both of which occur naturally in synovial fluid in soluble form, inhibiting TNF-α activity by competing with cell surface receptors for binding [5]. Similarly the two IL-1 receptors (IL-1R1and IL-1R2) also occur in soluble form in synovial fluid. These receptors are capable of binding IL-1, thus forming competition for cell surface receptors [6]. Additionally, a naturally occurring competitive inhibitor for IL-1 at the IL-1 receptor (IL-1 receptor antagonist IL-1RA) is also present in RA synovial fluid [7]. This member of IL-1 family binds itself to IL-1R1 without transducing a signal, thus blocking the receptor binding ability of IL-1. Results of work in animals have suggested a central role of TNF-α and IL-1 in the process of synovitis and joint destruction. Addition of exogenous TNF-α or IL-1 into experimental models of arthritis induces synovitis. Furthermore, mice transgenic for TNF-α, and mice with disregulated TNF-α production develop arthritis [8,9]. The treatment of murine models of arthritis with antibodies against TNF-α and IL-1 or with soluble TNF-α, receptor ameliorates or abrogates the disease [10,11].

## Methods

*Plumbago zeylanica *was collected in Paderu tribal area, Visakhapatnam, India and was authenticated by taxonomist Prof. B. Venkayya of the Botany department, Andhra University, India. A voucher specimen (4232) was deposited in herbarium of the Department of Botany, Andhra University.

### Preparation of ethanol extract of Plant

The alcoholic solubles (AS) were separated by vacuum filtration using a rotavapor. The resultant residue was freeze-dried to remove traces of alcohol. AS fraction was further separated to different fractions by using silica gel column chromatography using different organic solvents. Briefly activated silica gel was packed in a glass column (75 × 1 cm) up to 60 cm. The column was pre incubated with benzene and the above AS fraction (dissolved in 2 ml ethanol) was loaded on the top of the column and eluted with different organic solvents of increasing polarity^. ^The elution was carried out stepwise, with benzene, benzene: chloroform (1:1), chloroform, chloroform: ethylacetate (1:1), ethyl acetate: dichloromethane (1:1), dichloromethane, dichloromethane: ethanol (1:1) and ethanol. The vacuum evaporated fractions are further separated into six fractions and were designated as PZE-1, PZE-2, PZE-3, PZE-4, PZE-5 and PZE-6 (Plumbagin) with 5-30% ethyl acetate in benzene. These fractions were freeze dried and dissolved in olive oil for the current investigation.

### Animals

Male DBA/1 (H-2q) mice (8 weeks old) weighing around 30 gms were procured from the experimental animal facility of the National Institute of Immunology, New Delhi, India. The experimental protocol was approved by the Institutional ethics committee, Andhra University, Visakhapatnam, Andhra Pradesh, India.

### Chemicals and Materials

Bovine Type II collagen, Freund's Complete Adjuvant (FCA), Incomplete Freund's Adjuvant (IFA), Goat anti-mouse IgG, Bovine Serum Albumin (BSA), O-phenylenediamine (OPD), Hydrogen peroxide, RPMI-1640, Hank's balanced salt solution

(HBSS), Fetal Calf Serum (FCS), Ammonium Chloride and Streptavidin purchased from Sigma Chemical Company (St. Louis, MO, USA). Tritiated thymidine was supplied by Bhabha Atomic Research Centre (BARC), Mumbai, India. 96-well microtiter flat-bottom ELISA culture plates (Nunc, Denmark) were procured.

### Preparation of emulsified formulation of Type II collagen

Type II collagen (4 mg) was solubilized in 1 ml of 0.05 M acetic acid. Equal volumes of collagen solution were emulsified with FCA and IFA [12].

### Thin layer chromatography

Thin layer chromatography was performed to check the purity of the sample. Silica gel was manually coated onto the glass plate along with an adsorbent. Benzene: Chloroform (4:5) was used as a solvent. 5% alcoholic H_2_SO_4 _and Iodine were used to visualize the spots.

### ^1^H NMR Spectroscopic analysis of Plumbagin

Plumbagin fraction was analyzed on Gemini-200 MHz ^1^H NMR Spectrometer at Indian Institute of Chemical Technology, Hyderabad, India. The ^1^H NMR spectra of Plumbagin were recorded in CDCl_3 _with Trimethyl silane (TMS) as internal standard at room temperature.

### Statistical Analysis

Results were expressed as the average values with standard deviation (mean ± SD). Statistical significance was determined by Two-way analysis of variance (ANOVA) using SPSS (Statistical Package for the Social Sciences) software. Significance was set at p ≤ 0.05

### Immunization

The mice were (5 mice for each group) immunized with 0.1 ml of collagen with FCA (emulsion-A) by intradermal injection at the base of tail. On day 21, after primary immunization, mice were intradermally given a booster dose of collagen along with IFA (emulsion-B) at the same site.

### Treatment

Plumbagin was dissolved in olive oil at different concentrations (3.33, 6.66, 13.33 mg/kg body weight) and given intraperitoneally in a volume of 100 μl, weekly thrice for six weeks, starting from day 0 to day 42. Control mice received olive oil alone during this period. Indomethacin (3 mg/kg) was given intraperitoneally.

### Determination of antitype II collagen antibodies serum (ELISA method)

Retro orbital blood samples from arthritic mice were collected at 3, 5, 7 weeks after primary immunization and centrifuged at 2000 rpm for 10 min to get the required serum. Sera samples were diluted with saline. Microtitre plate (ELISA) was coated with type II collagen kept for overnight, and then

blocked with 50 μl of 2% BSA in PBS for 90 min at room temperature. The plate was washed 4 times with PBS (pH 7.5), 0.005% tween 20 (wash buffer) 50 μl of dilute sera was added to each well and then incubated for 60 min at room temperature. The plate was again washed 4 times with wash buffer. The peroxidase labelled rabbit anti-mouse IgG 50 μl/well (in 0.6 mol/lit NaCl, 0.26 mol/lit H_3_PO_4 _and 0.08 N NaOH, pH 9.6) was added to each well as the secondary antibody and incubated for 1 hour at room temperature. The plate was washed and 100 μl of 1.5 M citrate phosphate buffer containing OPD and 100 μl of H_2_O_2 _were added. The reaction was stopped after 5 min by adding 50 μl of 2N H_2_SO_4. _Colour developed was read at 490 nm using ELISA reader (Bio-rad model-550).

### T cell proliferation assay

For testing the effect of Plumbagin on con A induced T cell proliferation, On day 12 mice were sacrificed and their spleens were collected. T cells were isolated according to the procedure of Francis et al (1990) [13]. Briefly, the spleens were teased to get single cell suspension by incubating with 1 ml of 0.9% NH_4_Cl for 1 min at 37 °C, followed by arresting the activity of NH_4_Cl by adding RPMI twice the volume of the incubation mixture. The cells were washed and suspended in RPMI and incubated in a Petri dish coated with goat anti-mouse immunoglobulins at 1:1000 dilutions in PBS to remove the B cell population. The unbound cells were carefully separated and resuspended in FCS. The viability of cells was checked with tryphan blue exclusion [14]. These purified cells were employed in Ovalbumin induced T cell proliferation assay. T cell proliferation and cell cultures were carried out in sterile microtitre plates with 96 flat bottom wells. All the reagents used were filter sterilized using 0.22 nunc millipore filters prior to adding into the wells. For each individual assay, the cultures were done in triplicate, with each well containing 2 × 10^5 ^cells in 250 μl RPMI medium.

Cells were stimulated with Ovalbumin in absence or presence of different doses of PZE. Cultures were incubated for 72 h at 37 °C in a humified 5% CO_2 _modulator chamber. After 18 h the cultures were incubated with tritiated thymidine (0.5 μci/well).After incubation period, cells were harvested on glass fibre filters, using Nunc cell harvester and the thymidine incorporation was determined by liquid scintillation counter by adding 5 ml of scintillation fluid and 500 mg of 1,4 -bis{5-phenyl-2-oxazole}- benzene: 2,2'- p- phenylene-bis {5-phenyl- oxazole} per litre. The results were expressed as counts per minutes (cpm) and plotted against culture blanks, concentrations of Con A and Plumbagin.

### Assay of Cytokines

The estimation of cytokines was carried out using the culture supernatants from the *in vitro *spleen cell cultures for T cell assay. The spleen cells were collected from mice primed with different concentrations of Plumbagin. *In vitro *pulsing was done with Con A at a concentration of 1 μg. Spleen cell culture supernatants from unprimed mice treated with media and arthritic mice treated with media were used as controls. The supernatant for IL-2 estimation was collected after 24 hours culture while for IFN- γ estimation, it was collected after 72 hrs culture. The R&D cytokine assay (ELISA) kit was used for estimation. Briefly, the plate was coated with 100 μl of capture antibody (anti-mouse IFN-γ or IL-2) diluted in PBS (1 in 2000) and incubated overnight at room temperature. The plate was aspirated and washed thrice with wash buffer (0.5% tween-20 in PBS) using a multi channel pipette. Complete removal of liquid in each step was ensured and blotted against a clean paper after last wash. The wells were blocked with 300 μl of block buffer (1% BSA in PBS with 0.05% NaNO_3_) and incubated at room temperature for one hour. Again the plate was aspirated and washed. 100 μl of standards (recombinant mouse IFN-γ or IL-2) or culture supernatants primed with different concentrations of Plumbagin diluted in reagent diluents were added and plate was incubated for 2 hrs. After that the plate was aspirated, washed and incubated for 2 hrs with 100 μl of detection antibody (biotinylated goat anti-mouse IFN-γ or IL-2, diluted in reagent diluent).

Then 100 μl of diluted streptavidin (conjugated to horse raddish peroxidase, diluted in 1 in 1000 in reagent diluent) was added to each well and the plate was incubated for 20 min at room temperature. Exposing the plate to direct sunlight was avoided. The plate was aspirated, washed and incubated at room temperature for 20 min with substrate solution (1:1 mixture of H_2_O_2_) and trimethyl benzedene) of 100 μl per well. The plate was read at 450 nm after stopping the reaction with 50 μl/well of stopping solution (2N H_2_SO_4_).

## Results

**Figure 1:**
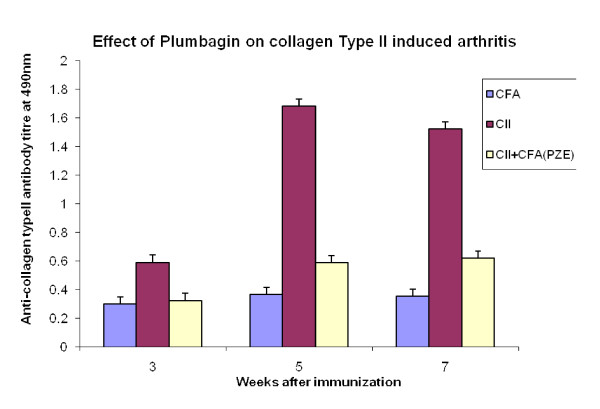
**Effect of Plumbagin on collagen type II induced arthritis**. Arthritis was induced in two groups (5 per each group) of mice by injecting collagen type II (CII) along with complete Freund's adjuvant (CII+CFA). Control group with CFA alone. Booster dose was given on day 21. One arthritic group was treated with Plumbagin (PZE-6) another treated with olive oil alone. Retroorbital sera were collected at 3, 5, 7 week intervals for the estimation of anti-type II collagen antibody (IgG) by ELISA. Bars represent means±SEM from n = 5. p-value≤0.05 was significant.

**Figure 2:**
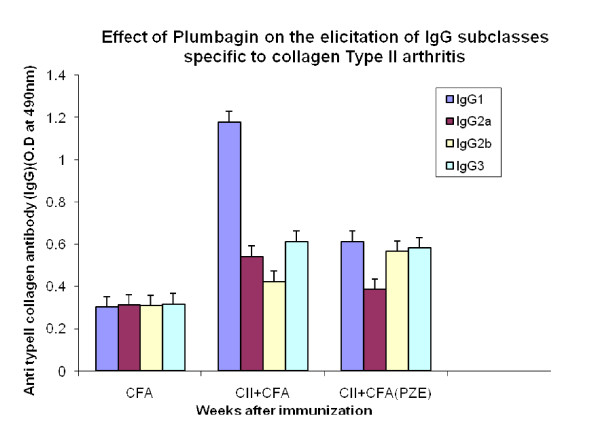
**Effect of Plumbagin on elicitation of Ig G subclasses specific to collagen type II arthritis**. Two groups of DBA/1 mice were immunized with 0.1 ml of bovine type II collagen along with CFA intradermally at the base of the tail. Booster dose was given on day 21. Test group was treated with Plumbagin. Control mice were treated with olive oil alone. Retroorbital sera were collected at 3, 5, 7 week intervals after primary immunization for the estimation of IgG subclasses by ELISA. p-value≤0.05 was significant.

**Figure 3:**
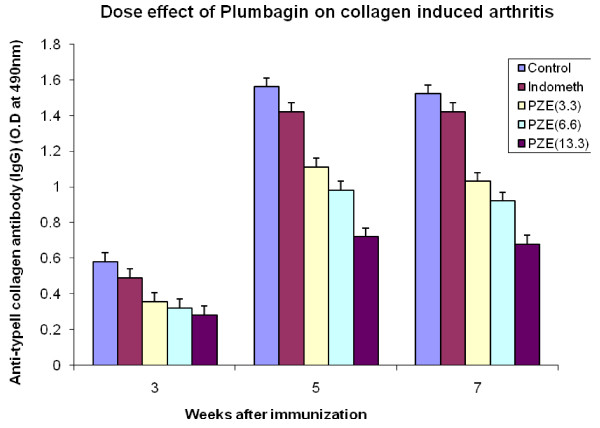
**Dose effect of Plumbagin on collagen Type II induced arthritis**. Five groups of DBA/1 mice were immunized with 0.1 ml of bovine type II collagen intradermally at the base of the tail. Booster dose was given 3 weeks after primary immunization. Test groups were treated with Plumbagin (3.3 mg/kg), (6.6 mg/kg) and (13.3 mg/kg) and Indomethacin (3 mg/kg) respectively. Control mice were treated with olive oil alone. Retroorbital sera were collected at 3, 5, 7 week intervals after primary immunization for the estimation of anti-type-II collagen antibody (IgG) by ELISA. p-value≤0.05 was significant.

**Figure 4:**
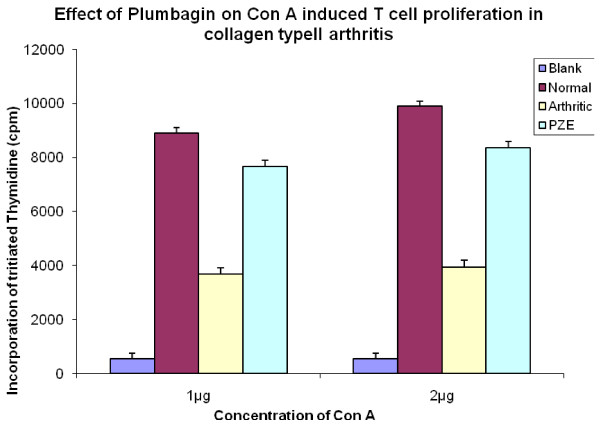
**Effect of Plumbagin on Con A induced T cell proliferation in collagen Type II induced arthritis**. Two groups of DBA/1 mice immunized with 0.1 ml of bovine type II collagen intradermally at the base of the tail. Booster dose was given on day 21. Normal group did not receive any injection. Test group was treated intraperitoneally with Plumbagin. Control group was treated with vehicle alone. At the end of the experiment mice were sacrificed, and Spleenic T lymphocytes isolated from arthritic mice, Plumbagin treated mice and normal mice were incubated with 1μg and 2μg of Con A and maintained the conditions similar to the previous culture experiments. Cultures were pulsed with 0.5 μci of radioactive thymidine per well and the incorporation of radioactivity was measured in scintillation counter after 18 hours of incubation. Native T cells without Con A stimulation served as blank. p-value≤0.05 was significant.

**Figure 5:**
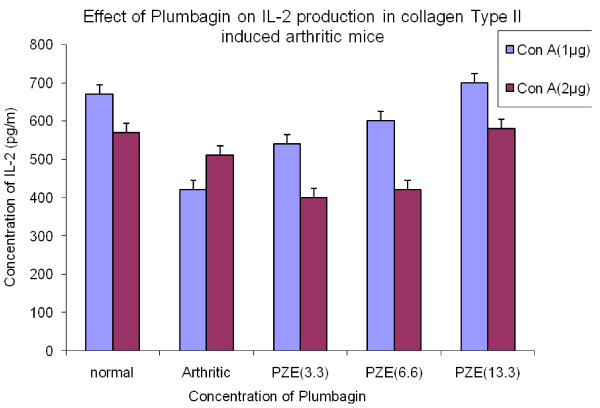
**Effect of Plumbagin on IL-2 production in collagen Type II induced arthritis mice**. Four groups of DBA/1 mice were immunized with 0.1 ml of bovine type II collagen intradermally at the base of the tail. Booster dose was given 3 weeks after primary immunization. Normal group did not receive any injection. Test groups were treated intraperitoeally with Plumbagin (3.3 mg/kg), (6.6 mg/kg) and (13.3 mg/kg) respectively. Control group was treated with vehicle alone. At the end of the experiment, mice were sacrificed and Spleenic T lymphocytes isolated from arthritic mice, PZE treated mice and normal mice were incubated with 1μg and 2 μg of Con A and maintained the conditions similar to the previous culture experiments. Cultures supernatants were collected and IL-2 production was estimated by ELISA. p-value≤0.05 was significant.

**Figure 6:**
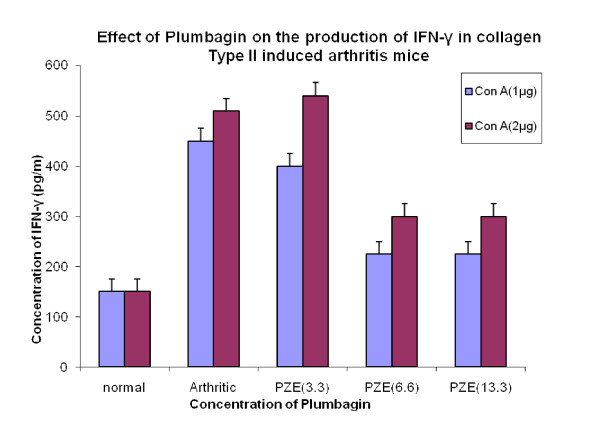
**Effect of Plumbagin on the production of IFN-γ in collagen Type II induced arthritis mice**. Four groups of DBA/1 mice were immunized with 0.1 ml of bovine type II collagen intradermally at the base of the tail. Booster dose was given 3 weeks after primary immunization. Normal group did not receive any injection. Test groups were treated intraperitoneally with Plumbagin (3.3 mg/kg), (6.6 mg/kg) and (13.3 mg/kg) respectively. Control group was treated with vehicle alone. At the end of the experiment, mice were sacrificed and Spleenic T lymphocytes isolated from arthritic mice, Plumbagin treated mice and normal mice were incubated with 1μg and 2μg of Con A and maintained the conditions similar to the previous culture experiments. Cultures supernatants were collected and IFN-γ production was estimated by ELISA. p-value≤0.05 was significant.

We have previously reported that PZE-6, ethyl acetate fraction of *Plumbago zeylanica *reduced the clinical score of the disease in CIA mice [15].Our findings in Figure 1 have shown that 3.3 mg/kg body weight of Plumbagin was significantly decreased the anti-collagen II antibodies determined by ELISA. All the four subclasses of type II collagen specific IgG (IgG1, IgG2a, IgG3 and IgG4) antibody responses in the Plumbagin treated groups were observed to be decreased throughout the experiment. Plumbagin (3.3 mg/kg body weight) significantly suppresses the IgG2a responses (Figure 2). Dose dependant suppression of antibody response to collagen type II was also observed in Plumbagin treated groups (3.33, 6.66 and 13.33 mg/kg) with the comparison of Indomethacin (3 mg/kg body weight) treated group as shown in the Figure 3. The results in Figure 4 revealed that the Con A induced T cell proliferative response of arthritic mice was found to be decreased significantly when compared to the T cell response of normal mice. However, the Plumbagin treatment of arthritic mice restored considerably the decrease of T cell proliferative response. The cytokine levels were estimated from the *in vitro *spleen T cell culture supernatants of arthritic mice primed with different concentrations of Plumbagin (3.33, 6.66 and 13.33 mg/kg body weight). Pulsing was done with 1μg concentration of Con A. the supernatants from 24 hr cultures were used for IL-2 estimation however for IFN- γ estimation, it was collected after 72 hrs. The results in Figure 5 demonstrated that with increase of Plumbagin concentration the IL-2 levels seems to be elevated to the normal levels in treated CIA mice. Consequently the down regulation of IFN- γ levels were observed in dose dependent manner in culture supernatants of T cells (Figure 6).

## Discussion

Trentham et al (1977) [12] induced arthritis in animal model by injecting type II collagen as an experimental model that is similar to human rheumatoid arthritis in several of its clinical and immunological manifestations. In this collagen induced arthritis (CIA) mouse model. Plumbagin (3.33 mg/kg body weight) could suppress the total anti collagen IgG responses significantly as determined by ELISA. Although all the four subclasses of IgG were decreased by Plumbagin treatment, IgG2a reduction seems to be more prominent. The disease susceptibility is also related to autoantibody isotype, Predominance of IgG2a isotype is associated with arthritis development as evidenced by the work of Watson & Townes, 1985 [16]. In addition, plumbagin treatment in arthritic mice stimulated the Con A induced T cell proliferation to the normal levels by stimulating IL-2 levels. T cells are believed to play a key role in the inflammatory response of the disease through the production of cytokines with different properties. Prophylactic treatment of adjuvant induced arthritic rats with a novel anti-inflammatory retinoids like 2,4,6,8 -nona tetraenoic acid, retinoid Ro236457, increased Con A induced IL-2 production by spleen cells [17]. HWA 486 is a very effective inhibitor of adjuvant arthritis which restored the depressed cellular immune reactivity [18]. The treatment with adjuvant induced arthritic rats with ethanolic root extract of *Plumbago zeylanica *on spleenic T cell proliferation compared with rats treated with known standard anti- inflammatory drugs [19]. Ethylacetate fraction of root extract of *Plumbago zeylanica *suppressed collagen induced arthritis in DBA/1 mice in dose dependent manner [15]. *N,N*-Dimethylglycine (DMG) and extracts from the New Zealand green-lipped mussel *Perna canaliculus *(Perna) were potent immunomodulators to modify ongoing immune and/or inflammatory responses. Extracts of *Perna canaliculus *derived from Tween-20, acid, or ethanol treatment were shown to significantly decrease the production of TNF-α, IL-1, IL-2, and IL-6 as well as hybridoma antibody production [20]. Recently reports suggested that anti-inflammatory and analgesic effect of Plumbagin through inhibition of nuclear factor-kappa B activation [21]. Plumbagin (3.33 mg/kg body weight) significantly suppresses the total IgG and IgG2a responses. More recently, an IL-15-Fcg2a fusion protein was generated that prevented the development of CIA and also blocked disease progression in an established disease model. The fusion protein was a construct of a point mutated IL-15 fused to the constant region of a murine IgG2a which binds to the IL-15R without inducing signalling, thereby blocking IL-15 [22]. From this study we can understand that the reduced IgG2a response by Plumbagin might influence the anti-inflammatory cytokine secretion by stimulating Th1 cells.

## Conclusion

The above study thus establishes the IgG2a suppression in Plumbagin treated CIA mice modulated the T cell proliferation through cytokine secretion. It may also have novel therapeutic role in the pathogenesis of rheumatoid arthritis. Modulation of cytokine levels in arthritic mice has also proved to be fruitful in the therapy of disease.

## Competing interests

The authors declare that they have no competing interests.

## Authors' contributions

AP carried out cell culture experiments, cytokine estimations and drafted the manuscript. RDN participated in the T cell proliferation assay. RRA participated in the design of study and its coordination. VS performed statistical analysis. All the authors read and approved the final manuscript.

## Pre-publication history

The pre-publication history for this paper can be accessed here:

http://www.biomedcentral.com/1472-6882/11/114/prepub
